# Vitamin D Receptor Genetic Polymorphisms Associate With a Decreased Susceptibility to Extremity Osteomyelitis Partly by Inhibiting Macrophage Apoptosis Through Inhibition of Excessive ROS Production *via* VDR-Bmi1 Signaling

**DOI:** 10.3389/fphys.2022.808272

**Published:** 2022-07-25

**Authors:** Xing-Qi Zhao, Hao-Yang Wan, Si-Ying He, Han-Jun Qin, Bin Yu, Nan Jiang

**Affiliations:** ^1^ Division of Orthopaedics and Traumatology, Department of Orthopaedics, Southern Medical University Nanfang Hospital, Guangzhou, China; ^2^ Guangdong Provincial Key Laboratory of Bone and Cartilage Regenerative Medicine, Southern Medical University Nanfang Hospital, Guangzhou, China

**Keywords:** vitamin D receptor (VDR), genetic polymorphism, ROS–reactive oxygen species, osteomyelitis (OM), macrophages

## Abstract

**Background:** Previous studies had reported that *vitamin D receptor* (*VDR*) gene polymorphisms were related to the development of several inflammatory disorders. However, potential links between such variations and the risk of developing a bone infection and underlying mechanisms remain unclear. This study aimed to analyze potential associations between *VDR* genetic variations and susceptibility to extremity osteomyelitis (OM) in a Chinese Han population and investigate potential mechanisms.

**Methods:** Between January 2016 and August 2020, altogether 398 OM patients and 368 healthy controls were genotyped for six *VDR* gene polymorphisms, including *ApaI* (rs7975232), *BsmI* (rs1544410), *FokI* (rs2228570), *TaqI* (rs731236), *GATA* (rs4516035), and *Cdx-2* (rs11568820) by the SNaPshot genotyping method. Then, male C57BL/6 mice were randomly divided into vitamin D–standard, –excess, –deficient, and –rescued groups. One week after making the model surgery, OM occurrence and severity were assessed using the bacterial count and histopathological staining. *In vitro*, phagocytosis, apoptosis, and bactericidal ability of macrophages were evaluated by overexpression or knockdown of VDR protein.

**Results:** Significant associations were found among rs7975232, rs1544410, and OM development by the recessive model (AA vs. AC + CC, *p* = 0.037, OR = 0.594), homozygous model (AA vs. CC, *p* = 0.033, OR = 0.575), and heterozygous model (CT vs. CC, *p* = 0.049, OR = 0.610), respectively. Patients with the AA genotype of rs7975232 had a relatively higher mean level of vitamin D than those with AC and CC genotypes (22.5 vs. 20.7 vs. 19.0 ng/ml). Similarly, patients with CT genotype of rs1544410 had a relatively higher mean vitamin D level than those with CC genotype (20.94 vs. 19.89 ng/ml). Outcomes of *in vivo* experiments showed that the femoral bacterial load of vitamin D–deficient mice was highest among different vitamin D dose groups, with the most severe histopathological features of infection, and vitamin D supplementation partly reversed the changes. While *in vitro* experiment results revealed that active vitamin D promoted phagocytosis and sterilization of macrophages and inhibited apoptosis during infection. Reactive oxygen species (ROS) inhibitor inhibited apoptosis of macrophages induced by bacterial infection. Active vitamin D inhibited excessive ROS production in macrophages *via* the VDR-Bmi1 signaling pathway.

**Conclusion:** In this Chinese cohort, *ApaI* and *BsmI* are associated with a decreased risk of OM development by influencing serological vitamin D level, the latter of which reduced macrophage apoptosis with inhibition of excessive ROS production *via* the VDR-Bmi1 signaling pathway.

## Introduction

Bone infection, also known as osteomyelitis (OM), is a progressive inflammatory process leading to the destruction and necrosis of the bone (Birt et al., 2017). According to different pathogenic mechanisms, this disorder can occur following either an exogenous approach or hematogenous spread. The former refers to direct contamination of osseous tissue secondary to open fracture, orthopedic surgery with or without implants, or local spread from contiguous sites of infection (Arshad et al., 2021), with fracture-related infection (FRI) as a representative. While hematogenous OM means the nidation of microorganisms present in the systemic circulation into the bone. Despite different etiologies, in the present, OM still represents one of the most challenging disorders for orthopedic surgeons, not only owing to non-specific symptoms of the patients but also attributing to the unsatisfying clinical efficacy. Currently, OM brings both physical and psychological pressures to the patients (Tseng et al., 2014; Hung et al., 2020). Even in some patients with long-term skin ulcers and sinus drainage, malignant transformation of squamous cell carcinoma is not seldom (Jiang et al., 2020a). Therefore, how to decrease the OM incidence is of great clinical significance, which is built on a comprehensive understanding of its pathogenesis.

It is known that OM occurrence is associated with both external and internal factors. While most previous studies analyzed its pathogenesis from the perspective of environmental factors. Recently, growing evidence has shown that single nucleotide polymorphism (SNP) also plays an important role in OM development. Several SNP sites have been found to be associated with the risk of OM development, such as rs16944, rs2234663, rs1143627, rs4251961, and rs1800796 (interleukin, *IL* genes) (Alves De Souza et al., 2017; Jiang et al., 2020b), rs45567233 (cathepsin G, *CTSG* gene) (Pérez-Is et al., 2019), rs1799750, and rs1144393 (matrix metalloproteinase-1, *MMP-1* gene) (Kong et al., 2017), implying SNPs participate in OM pathogenesis.

Vitamin D involves an extensive variety of biological processes, such as bone metabolism, regulation of cell proliferation and differentiation, and modulation of the immune response. With respect to the potential role of vitamin D in osteoarticular infection, a previous study reported that vitamin D deficiency might increase the risk of prosthetic joint infection (PJI) after primary arthroplasty (Maier et al., 2014). Also, an *in vivo* experiment showed vitamin D deficiency resulted in increased bacterial burden, which could be reversed with preoperative repletion of vitamin D. Meanwhile, vitamin D deficiency also significantly reduced the activated tissue macrophage recruitment in the infection site (Hegde et al., 2017a). These suggest that vitamin D may have a protective effect in case of bone and joint infection; however, the detailed mechanisms remain largely unclear.

Vitamin D mediates its function by binding to the vitamin D receptor (VDR), encoded by the *VDR* gene, which is highly polymorphic. The most frequently reported *VDR* genetic variations include *ApaI* (rs7975232), *BsmI* (rs1544410), *FokI* (rs2228570), *TaqI* (rs731236), *GATA* (rs4516035), and *Cdx-2* (rs11568820). Previous studies found that these SNPs are associated with the susceptibilities to many inflammatory disorders, such as COVID-19 (Apaydin et al., 2021), dengue (Singh et al., 2021), and community-acquired pneumonia (Awasthi et al., 2021). With regard to OM, our previous study found that *TaqI* (rs731236) and *FokI* (rs2228570) increased the risks of developing chronic OM in the Chinese population (Jiang et al., 2016). However, the sample size was limited. More important, potential relationships between different genotypes and serum vitamin D levels were not explored, and thus, the effects of genotype on vitamin D level in OM patients is still unknown. While in a subsequent study (Zhao et al., 2022), positive links were found between *VDR* gene polymorphisms and susceptibilities to FRI, and definite relationships were also obtained between different genotypes and serum vitamin D levels.

Based on recent literature and our previous outcomes, we speculated that one underlying mechanism that *VDR* SNPs involve in the OM development rests with the possibility that such genetic variations may influence serological vitamin D levels, the latter of which may affect VDR protein expression. Different VDR levels in macrophages may influence macrophage functions, involved in the pathogenesis of OM.

## Materials and Methods

### Definition, Inclusion and Exclusion Criteria, and Study Registration

The first part of this study was designed as a case-control investigation, with comparisons conducted between OM patients and healthy controls. OM is defined as bone infection with or without the surrounding soft tissue infection following an exogenous approach and hematogenous spread (Arshad et al., 2021). OM diagnosis was established based on any of the following confirmatory criteria, including wound breakdown or sinus or fistula connecting the bone or the implant, positive pathogen culture, and histology test outcomes (McNally et al., 2020). Participants included in the patient group were those who had been diagnosed with OM (following exogenous approach and hematogenous spread) in the Southern Medical University Nanfang Hospital, between 1 January 2016 and 31 August 2020. Patients who were receiving vitamin D supplementation, or received vitamin D supplementation within 1 month or relevant diet were excluded. Eligible participants of the control group were healthy individuals after thorough examinations in our hospital. All the included participants signed the informed consent and this study was in accordance with the tenets of the Helsinki declaration. This study had been approved by the medical ethics committee of the hospital (NFEC-2019-087).

### DNA Extraction and Single Nucleotide Polymorphism Genotype

Ethylene diamine tetraacetic acid (EDTA) prepared peripheral blood samples (5 ml for each) were collected and stored at −80°C. Then, the genomic DNA of each sample was extracted from the peripheral blood leukocytes according to instructions of the Flexi Gene-DNA Kit (Qiagen, Valencia, CA, United States). The six tag SNPs of the *VDR* gene (rs7975232, rs1544410, rs2228570, rs731236, rs4516035, and rs11568820) were genotyped using the Multiplex SNaPshot system (Applied Biosystems, Foster City, United States). The forward (F), reverse (R), and extension primers used for polymerase chain reaction (PCR) and extension reactions of the six SNPs were listed in Table 1. Detailed procedures of SNaPshot genotyping method were described previously (Jiang et al., 2016).

**TABLE 1:** PCR primers and extension primers of the six *VDR* genetic polymorphisms.

SNP	PCR primers	Extension primers
rs7975232	F: 5′-GGC​ACG​GGG​ATA​GAG​AAG​AA-3′	5′-CTG​ACT​GAC​TGA​CTG​ACT​CAC​AGG​AGC​TCT​CAG​CTG​GGC-3′
	R: 5′-GCA​CGG​AGA​AGT​CAC​TGG​A-3′	
rs1544410	F: 5′-GTG​CAG​GCG​ATT​CGT​AGG-3′	5′-TGG​GGC​CAC​AGA​CAG​GCC​TGC-3′
	R: 5′-ACC​ATC​TCT​CAG​GCT​CCA​AA-3′	
rs2228570	F: 5′-GCA​CTG​ACT​CTG​GCT​CTG​A-3′	5′-CTG​ACT​GAC​TGA​CTG​ACT​GAC​TGA​CTC​TTG​CTG​TTC​TTA​CAG​GGA-3′
	R: 5′-GCC​TTC​ACA​GGT​CAT​AGC​ATT​G-3′	
rs731236	F: 5′-TGG​TGG​GAT​TGA​GCA​GTG​A-3′	5′-GCA​GGA​CGC​CGC​GCT​GAT-3′
	R: 5′-GAA​GGA​GAG​GCA​GCG​GTA-3′	
rs4516035	F: 5′-CCT​CTT​CTT​AGA​ACT​CAC​TGT​GC-3′	5′-TCC​TTT​AGC​CAG​GGA​AGA-3′
	R: 5′-CCT​TGT​CCC​TCT​GAG​CCA​T-3′	
rs11568820	F: 5′-AGA​ACA​TCT​TTT​GTA​TCA​GGA​ACT-3′	5′-CTG​ACT​GAC​TGA​CTG​ACT​GAC​TGA​CTC​CTG​AGT​AAA​CTA​GGT​CAC​A-3′
	R: 5′-AAT​GTA​AGA​AGC​TGT​AGC​AAT​GAA-3′	

### Animal and Vitamin D Protocols

Eight-week-old male C57BL/6 mice were used in the *in vivo* experiments, which were originally obtained from the Animal Center of our hospital, bred, and maintained under specific pathogen-free conditions at an American Association for the Accreditation of Laboratory Animal Care–accredited animal facility at Southern Medical University. The mice were kept three mice per cage and fed either a standard Ain-93G diet (1,000 IU vitamin D per kg of feed), a vitamin D–excess diet (5,000 IU vitamin D per kg of feed), or a vitamin D–deficient diet (0 IU vitamin D added) with access to bottled water. Veterinary staff carried out daily evaluations. All the animal experiments were approved by Southern Medical University Nanfang Hospital Animal Ethics Committee and were conducted in accordance with the relevant ethical principles and guidelines set by the Animal Welfare Act and the NIH Guide for Care and Use of Laboratory Animals.

The mice were randomized to receive either a standard Ain-93G (*n* = 9), vitamin D–excess (*n* = 9) or vitamin D–deficient (*n* = 18) diet. Mice were fed the chosen diet for 10 weeks before surgery to ensure vitamin D sufficiency or deficiency. Three days before the surgery, the “rescued” group (*n* = 9) was created from the mice being fed a deficient diet by injecting 80 ng of vitamin D (Sigma-Aldrich) intraperitoneally, as previously described (Hegde et al., 2017b); these mice were also switched over to a vitamin D–excess diet.

### Bacteria Strain and Cultures

*Staphylococcus aureus* (*S. aureus*) strain ATCC 25923 provided by the Infectious Diseases Department of our hospital was verified by PCR amplification. *S. aureus* was cultured in tryptic soy broth (TSB) (BD Biosciences, San Jose, CA, United States) at 37°C in a shaking incubator at 200 rpm overnight for 16 h. Bacteria in the log phase were harvested by centrifugation at 3,000 rpm for 10 min, resuspended in sterile phosphate-buffered saline (PBS), and washed for three times. *S. aureus* concentration was determined by measuring the absorbance at 600 nm, combined with serial dilution on tryptone soy agar (TSA) (BD Biosciences, San Jose, CA, United States) containing 5% sheep blood. Colony-forming units (CFUs) were verified after overnight culture of plates.

### Surgical Procedures

Surgical procedures of implant-associated infection (IAI), which resembled OM, were modified based on previous work (Bernthal et al., 2010; Hegde et al., 2017b; Wang et al., 2017). Briefly, mice (18 weeks old) were anesthetized *via* intraperitoneal injection of tribromoethanol (1.25%). The skin around the right knee joint was prepared and disinfected. After the femoral intercondylar notch was located, a disposable insulin syringe with a 29-gauge needle (KRUUSE, China) was percutaneously inserted into the femoral intramedullary canal. The needle position was confirmed *via* X-ray, followed by manual reaming. An inoculum of 1 × 10^2^ CFUs of *S. aureus* in 2 µl of normal saline solution was pipetted into the intramedullary canal. After a stainless-steel acupuncture needle (femoral prosthesis, length 10 mm, diameter 0.2 mm; Zhongyan Taihe Medical Instrument, Beijing, China) was surgically placed in a retrograde fashion, its tail end was cut off after withdrawal by 2 mm. Passive movement of the mouse knee joint was maintained to ensure that the needle had completely entered the intramedullary canal. X-rays were retaken to check the position of the femoral prosthesis. No antibiotics were given throughout the study period. One week after surgery, the animals were examined for signs of infection.

### Implant-Associated Infection Occurrence and Severity Evaluation

Part of the femurs was used for bacterial load evaluation, with the rest for severity evaluation. The bacterial load (CFUs) at the right femur was used to assess the occurrence of IAI. After the femoral prosthesis removal, the right femur was collected and weighed, placed in 2 ml of sterile PBS, and homogenized for 5 min. The resulting homogenized solution was diluted with 4°C TSB and plated in triplicate for overnight culture to achieve tissue bacterial load. As for the infection severity evaluation, the femurs were decalcified in 10% ethylenediaminetetraacetic acid (pH 8.0) at room temperature for 1 week, dehydrated through a graded ethanol series, and embedded in paraffin. Sections (4 μm) were cut longitudinally and processed for staining with hematoxylin and eosin (H&E) staining. Smeltzer’s scoring (Smeltzer et al., 1997) was used to evaluate the severity of IAI by two blinded observers according to H&E staining results. Each section was assigned a score, which is the sum of intraosseous acute inflammation (0–4), intraosseous chronic inflammation (0–4), periosteal inflammation (0–4), and bone necrosis (0–4).

### Immunofluorescence Analysis

For immunofluorescence analysis, sections were blocked with 10% goat serum (Vector Laboratories, Burlingame, CA, United States) at room temperature for 1 h and incubated with primary antibody against *S. aureus* (ab20920, Abcam) and macrophages (Anti-F4/80 antibody, RT1212, HuaBio) overnight at 4°C. The secondary antibodies, Alexa Fluor 594-conjugated goat anti-rabbit IgG (Proteintech, Wuhan, China) and FITC-conjugated goat anti-rat IgG (AP183F, Sigma-Aldrich) were used to visualize signals, and the sections were subsequently stained with 4′,6-diamidino-2-phenylindole (DAPI; Vector Laboratories, Burlingame, CA, United States) in the dark. Images were acquired using a fluorescence microscope (Olympus BX63). Quantitative analysis was conducted in a blinded fashion using ImageJ software (ImageJ 1.51j8).

### Activated Vitamin D–Mediated Vitamin D Receptor Overexpression in Macrophages

After stimulated adherence, the original medium of macrophages was discarded and replaced with a complete medium containing active vitamin D (Sigma-Aldrich) concentrations of 0 M, 10^−9^ M, 10^−8^ M, and 10^−7^ M. After 24 h of culture, the medium was discarded and washed three times with PBS to obtain macrophages and extract total proteins. Western blotting (WB) was used to detect VDR protein expression. If other *in vitro* experiments were needed, a new medium was replaced with the same active vitamin D concentration.

### siRNA-Mediated Vitamin D Receptor Knockdown in Macrophages

The VDR siRNAs were purchased from Ribobio Biology, with siVDR1 (genOFFTM st-h-VDR_001) targeting AGC​GCA​TCA​TTG​CCA​TAC​T, siVDR2 (genOFFTM st-h-VDR_002) targeting GTCAGTTACAGCATCCA AA, and siVDR3 (genOFFTM st-h-VDR_003) targeting CCC​ACC​ATA​AGA​CCT​ACG​A. Macrophages were transfected with 1.25 μl 20 μM siRNA using ribo*FECT*™ CP transfection reagent. After 24 h, macrophages were harvested and protein expressions were detected.

### *In Vitro* Macrophage Infection Model

Human leukemia monocytic cell line (THP-1, ATCC TIB-202) and mouse macrophage cell line (RAW264.7, ATCC TIB-71), infected with *S. aureus* were used to evaluate the effects of vitamin D on macrophage functions. First, the number of macrophages was adjusted to 2 × 10^6^ cells/ml. After culturing in a 24-well plate (37°C, 5% CO_2_) for 24 h, the corresponding irritants were added for 24 h, and then *S. aureus* with multiple of infection (MOI) of 10 was added. Immunofluorescence was performed 15, 30, and 60 min after adding bacteria to observe the phagocytic and bactericidal ability of macrophages. Briefly, at the set point in time, cells were washed with PBS three times, then fixed with 4% formaldehyde, followed by perforation with 0.5% Triton X-100. Sequentially, infected macrophages were blocked with 5% bovine serum albumin (BSA) to prevent nonspecific conjunction and incubated with primary antibody against *S. aureus* at 4°C overnight. Then the secondary antibody, Alexa Fluor 594-conjugated goat anti-rabbit IgG was used to visualize *S. aureus* signals, subsequently stained with phalloidin and DAPI in the dark to visualize F-Actin (cytoskeleton) and nucleus signals, respectively. The cells were detected using fluorescent microscopy eventually. Meanwhile, after the bacteria were added for 1 h, Triton X-100 was used to break the cell membrane, and the adherent cells were blown into the supernatant. The collected supernatant was diluted with 4°C PBS and plated in triplicate for overnight culture to obtain the bactericidal ability of macrophages with different irritants.

### Apoptosis and Intracellular Reactive Oxygen Species Production Detections

Caspase-3/7 and propidium iodide (PI) fluorescent labeling dye (ribo*APO*™) were used to detect macrophage apoptosis and necrosis after bacterial infection according to the instructions. Fluorescence of cells was detected through fluorescent microscopy (Zeiss, Axio Imager. D2). Peroxide-sensitive dye H2DCF-DA was used to detect the level of reactive oxygen species (ROS) generation according to the instructions. The fluorescence of cells was detected through fluorescent microscopy.

### Western Blotting

After appropriate stimulation, equal amounts of protein (30 μg) extracted from macrophages were separated by sodium dodecyl sulfate-polyacrylamide gel electrophoresis and transferred to nitrocellulose membranes. The membranes were blocked in Tris-buffered saline Tween-20 containing 5% BSA for 1 h and then incubated overnight at 4°C with primary antibodies against B-cell lymphoma-2 (BCL-2), BCL2-associated X (BAX), pro-casepase-3, casepase-3, and GAPDH. After incubation with a secondary antibody for 1 h at room temperature, the membranes were visualized using the chemiluminescence method. Antibodies against BCL-2 (ET1603-11; Dilution: 1:1,000), BAX (ET603-34; Dilution: 1:1,000), pro-casepase-3 (ET1608-64; Dilution: 1:1,000), casepase3 (ET1602-47; Dilution: 1:1,000), and GAPDH (ET1601-4; Dilution: 1:3,000) were purchased from HuaBio (Hangzhou, China).

### Statistical Analysis

Statistical analysis was conducted using the Statistical Product and Service Solutions software (version 17.0, SPSS Inc., Chicago, IL, United States). All the reported values were 2-sided with a *p* value of lower than 0.05 as a statistical significance. For the case-control study, distributions of continuous variables were first assessed for normality using the Kolmogorov–Smirnov test. Then, the data were presented as mean ± standard deviation (SD) or median with interquartile range (IQR) based on data distribution. For normally distributed data, the Student’s test or one-way analysis of variance (ANOVA) test was used to compare differences between two groups or among three groups. Otherwise, the Mann–Whitney test or Kruskal–Wallis test was applied, respectively. When using an ANOVA analysis, a homogeneity test of variances was also conducted. When the homogeneity of variances assumption was met, the LSD method was used for post hoc multiple comparisons. Otherwise, Welch’s ANOVA test and Dunnett’s T3 method were applied for the whole and post-hoc multiple comparisons, respectively. The genotype distributions of the healthy controls were tested for confirmation of Hardy–Weinberg equilibrium (HWE) using the chi-square test. The chi-square test or Fisher exact test was used to compare the genotype distributions and frequencies of mutant allele, and the four genetic models (dominant, recessive, homozygous, and heterozygous models), with corresponding odds ratios (ORs) and 95% confidence intervals (CIs) between the patients and healthy controls. For the *in vivo* and *in vitro* experiments, results were expressed as mean ± standard error (SE). Statistical tests included two-tailed Student’s *t*-test, ANOVA followed by Tukey’s multiple comparison test for parametric statistics, and Kruskal–Wallis test followed by Dunn’s multiple comparisons for nonparametric statistics.

## Results

### Demographics and Characteristics of the Included Osteomyelitis Patients

Altogether 398 OM patients (312 males and 86 females) and 368 healthy controls (268 males and 100 females) were included, with no statistical differences regarding sex ratio (3.6 vs. 2.7, *p* = 0.073) and median age (45 vs. 46 years, *p* = 0.34) between the two groups. Among the 398 patients, 336 patients were as FRI and 62 patients with infection following hematogenous spread. There were 196 patients with infection on the left body side, with 195 on the right and 7 on bilateral sides. Among the 320 cases with a single lesion of infection, the most frequent site was tibia (174 cases), followed by the femur (56 cases) and calcaneus (33 cases), respectively. The total positive rate of sample culture was 57.5% (158/275), with monomicrobial infection accounting for 68.4% (108/158). The top frequently pathogens were *S. aureus* (54), *Pseudomonas aeruginosa* (Singh et al., 2021), *Enterobacter cloacae* (Maier et al., 2014), *Staphylococcus epidermidis* (Pérez-Is et al., 2019), and *Enterococcus faecalis* (Jiang et al., 2020a).

### Hardy–Weinberg Equilibrium Test Outcomes of the Healthy Controls

All the genotyped six *VDR* genetic variations were in HWE among the healthy controls (*p* = 0.55 of rs7975232; *p* = 0.77 of rs1544410; *p* = 0.08 of rs2228570; *p* = 0.21 of rs731236; *p* = 0.63 of rs4516035; *p* = 0.18 of rs11568820), demonstrating participants of the controlled group were representative.

### Associations Between Vitamin D Receptor Genetic Polymorphisms and Susceptibilities to Osteomyelitis

Significant links were found between rs7975232 and risk of OM development by recessive model (OR = 0.594, 95% CI 0.362–0.973, *p* = 0.037) and homozygous model (OR = 0.575, 95% CI 0.344–0.961, *p* = 0.033), suggesting that people with AA genotype were in a lower risk to develop OM. In addition, a significant association was identified between rs1544410 and susceptibility to OM by the heterozygous model (OR = 0.610, 95% CI 0.371–1.002, *p* = 0.049), demonstrating CT genotype may have a protective effect against OM. Although no statistical difference was obtained, the potential relationship may exist between rs2228570 and the risk of OM development by the heterozygous model (OR = 0.706, 95% CI 0.487–1.023, *p* = 0.065), implying that people with AG genotype may in a lower risk of OM development (Table 2).

**TABLE 2:** Genotype distribution, allele frequency, and genetic models of the six *VDR* genetic variations between OM patients and healthy controls.

SNP	Item	Patients	Controls	*p* values	OR (95%CI)	
*ApaI* rs7975232	Genotype (*n*, %)	AA	29 (7.3)	43 (11.7)	0.102	
		AC	173 (43.5)	158 (42.9)		
		CC	196 (49.2)	167 (45.4)		
	Allele frequency	A vs. C	231/565	244/492	0.081	0.824 (0.664–1.024)
	Dominant model	AA + AC vs. CC	202/196	201/167	0.284	0.856 (0.644–1.138)
	Recessive model	AA vs. AC + CC	29/369	43/325	**0.037**	0.594 (0.362–0.973)
	Homozygous model	AA vs. CC	29/196	43/167	**0.033**	0.575 (0.344–0.961)
	Heterzygous model	AC vs. CC	173/196	158/167	0.649	0.933 (0.692–1.257)
*BsmI* rs1544410	Genotype (*n*, %)	TT	1 (0.3)	1 (0.3)	0.144	
		CT	29 (7.3)	42 (11.4)		
		CC	368 (92.4)	325 (88.3)		
	Allele frequency	T vs. C	31/765	44/692	0.059	0.637 (0.398–1.021)
	Dominant model	TT + CT vs. CC	30/368	43/325	0.051	0.616 (0.378–1.005)
	Recessive model	TT vs. CT + CC	1/397	1/367	0.956	0.924 (0.058–14.833)
	Homozygous model	TT vs. CC	1/368	1/325	1.000	0.883 (0.055–14.176)
	Heterzygous model	CT vs. CC	29/368	42/325	**0.049**	0.610 (0.371–1.002)
*FokI* rs2228570	Genotype (*n*, %)	GG	104 (26.1)	103 (28.0)	0.154	
		AG	200 (50.3)	199 (54.1)		
		AA	94 (23.6)	66 (17.9)		
	Allele frequency	G vs. A	408/388	405/331	0.140	0.859 (0.703–1.051)
	Dominant model	GG + AG vs. AA	304/94	302/66	0.053	0.707 (0.497–1.006)
	Recessive model	GG vs. AG + AA	104/294	103/265	0.563	0.910 (0.662–1.252)
	Homozygous model	GG vs. AA	104/94	103/66	0.105	0.709 (0.468–1.075)
	Heterzygous model	AG vs. AA	200/94	199/66	0.065	0.706 (0.487–1.023)
*TaqI* rs731236	Genotype (*n*, %)	GG	0 (0.0)	2 (0.5)	0.23	
		AG	28 (7.0)	32 (8.7)		
		AA	370 (93.0)	334 (90.8)		
	Allele frequency	G vs. A	28/768	36/700	0.179	0.709 (0.428–1.174)
	Dominant model	GG + AG vs. AA	28/370	34/334	0.264	0.743 (0.441–1.252)
	Recessive model	GG vs. AG + AA	0/398	2/366	0.230	1.005 (0.998–1.013)
	Homozygous model	GG vs. AA	0/370	2/334	0.226	1.006 (0.998–1.014)
	Heterzygous model	AG vs. AA	28/370	32/334	0.381	0.790 (0.466–1.340)
*GATA* rs4516035	Genotype (*n*, %)	CC	0 (0.0)	0 (0.0)	0.168	
		CT	29 (7.3)	18 (4.9)		
		TT	369 (92.7)	350 (95.1)		
	Allele frequency	C vs. T	29/767	18/718	0.174	1.508 (0.830–2.739)
	Dominant model	CC + CT vs. TT	29/369	18/350	0.168	1.528 (0.834–2.801)
	Recessive model	CC vs. CT + TT	0/398	0/368	N/A	N/A
	Homozygous model	CC vs. TT	0/369	0/350	N/A	N/A
	Heterzygous model	CT vs. TT	29/369	18/350	0.168	1.528 (0.834–2.801)
*Cdx-2* rs11568820	Genotype (*n*, %)	TT	71 (17.8)	69 (18.8)	0.641	
		CT	193 (48.5)	166 (45.1)		
		CC	134 (33.7)	133 (36.1)		
	Allele frequency	T vs. C	335/461	304/432	0.757	1.033 (0.843–1.265)
	Dominant model	TT + CT vs. CC	264/134	235/133	0.473	1.115 (0.828–1.501)
	Recessive model	TT vs. CT + CC	71/327	69/299	0.745	0.941 (0.652–1.357)
	Homozygous model	TT vs. CC	71/134	69/133	0.920	1.021 (0.678–1.537)
	Heterzygous model	CT vs. CC	193/134	166/133	0.376	1.154 (0.840–1.585)

Bold values represent statistical signicance. OM, osteomyelitis; OR, odds ratio; CI, confidence interval; N/A, not available.

### Serum Levels of Total Vitamin D Among Different Genotypes of rs7975232, rs1544410, and rs2228570 in the Osteomyelitis Patients

**FIGURE 1:**
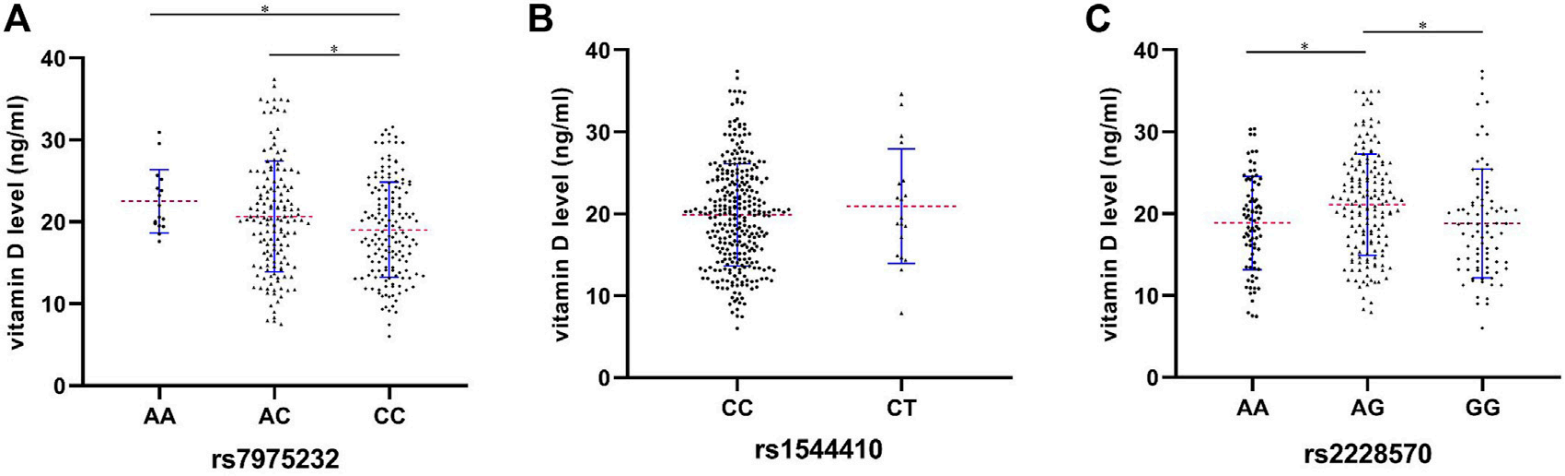
Preoperative serum levels of vitamin D among different genotypes of rs7975232 **(A)**, rs1544410 **(B)**, and rs2228570 **(C)** in the OM patients. *p < 0.05, **p < 0.01, and ***p < 0.001.

As shown in Figure 1, a significant difference was found regarding the mean vitamin D level among different genotypes of rs7975232 (*p* = 0.018), revealing the mean level of vitamin D of the AA genotype as the highest among the three groups (AA vs. AC vs. CC: 22.5 vs. 20.7 vs. 19.0 ng/ml) (Figure 1A). With regard to rs1544410, although no statistical difference was found between CC and CT genotypes (*p* = 0.477), the mean vitamin D level of the CT genotype was higher than that of the CC genotype (20.9 vs. 19.9 ng/ml) (Figure 1B). As mentioned previously, people with the AG genotype of rs2228570 may be at a lower risk of OM development though no statistical difference was obtained based on the outcomes of dominant and heterozygous models. Interestingly, the mean vitamin D level of the AG genotype was the highest among the three genotypes (AA vs. AG vs. GG: 18.9 vs. 21.1 vs. 18.8 ng/ml) (Figure 1C).

### Vitamin D Supplementation Inhibited the Risks of Implant-Associated Infection Occurrence and Severity

**FIGURE 2:**
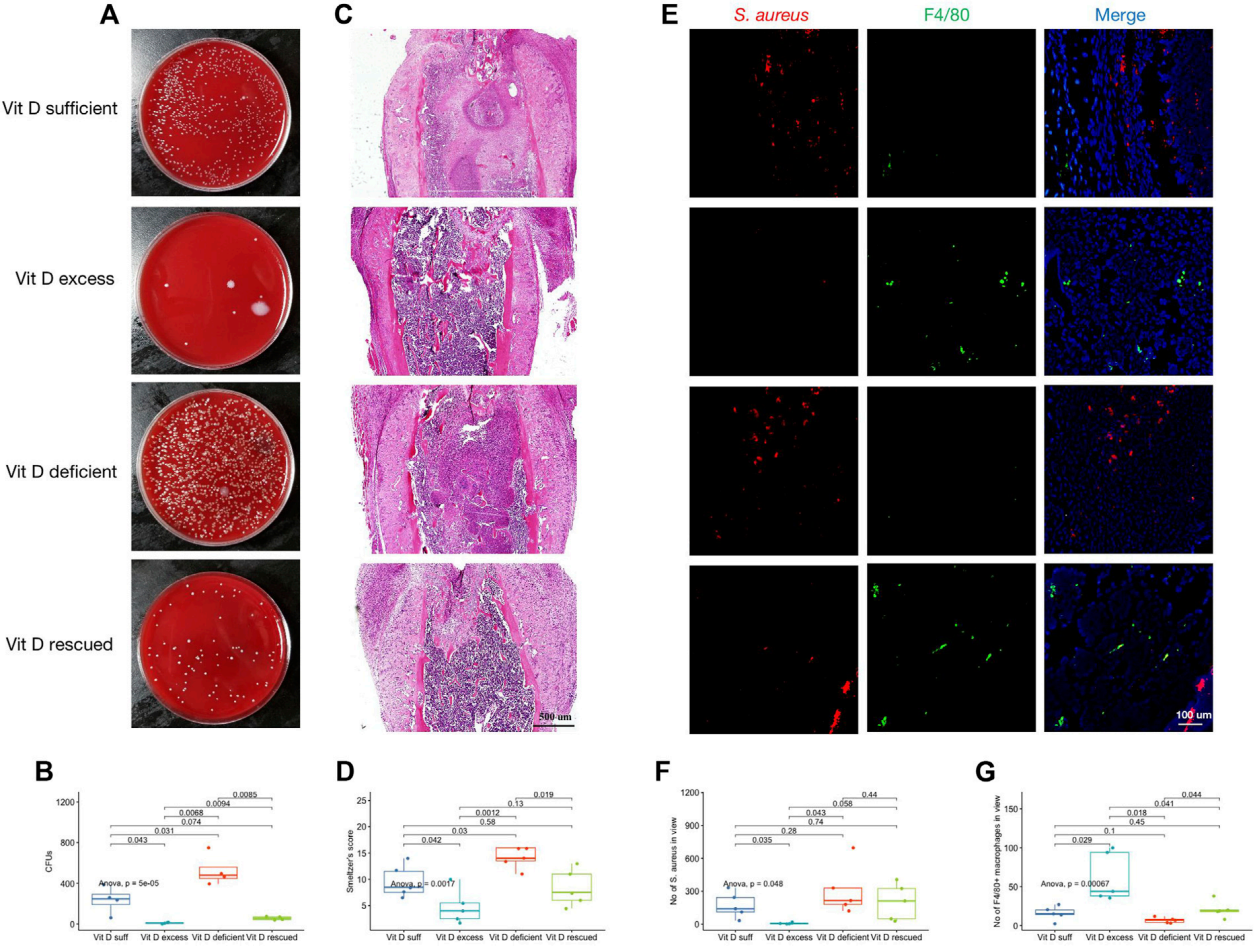
Vitamin D supplementation inhibited the risks of IAI occurrence and severity. **(A)** After the femoral implant was removed, the infected femur was ground and made into a homogenized solution. Representative images after coating the plate with the homogenized solution overnight. **(B)** CFUs of homogenized solution in each group. **(C)** Representative images of H&E staining. Magnification, ×40. **(D)** Smeltzer’s score. Mean value of two experienced reviewers’ scores. **(E)** Double immunofluorescence staining for *S. aureus* (red) and F4/80^+^ macrophages (green). Magnification, ×200. **(F)** Count of *S. aureus* in view. **(G)** Count of F4/80^+^ macrophages in view.

After removing the implants, the infected femur was weighed, ground, dissolved, and homogenized in PBS at 4°C aseptically. The bacterial growth after the homogenized solution was coated on the blood plate overnight, which was shown in Figure 2A. CFUs of homogenized solution in each group were drawn in Figure 2B. *S. aureus* load was significantly higher in the vitamin D–deficient group than that of vitamin D–excess and vitamin D–rescued groups. The trend of Smeltzer’s scores was consistent with that of the bacterial load (Figures 2C,D). These results suggested that vitamin D deficiency increased the risks of IAI occurrence and severity. In addition, *S. aureus* and macrophages were labeled on femoral tissue sections (Figure 2E). The number of positive bacterial markers was inconsistent with the results of the coating plate after grinding of the femoral tissue (Figure 2F). An interesting phenomenon was also noted that the number of F4/80 positive macrophages in vitamin D–deficient mice was significantly lower after IAI modeling, however, it was significantly improved after vitamin D was remedied (Figure 2G).

### Vitamin D Enhanced Macrophage Phagocytosis and Bactericidal Ability

**FIGURE 3:**
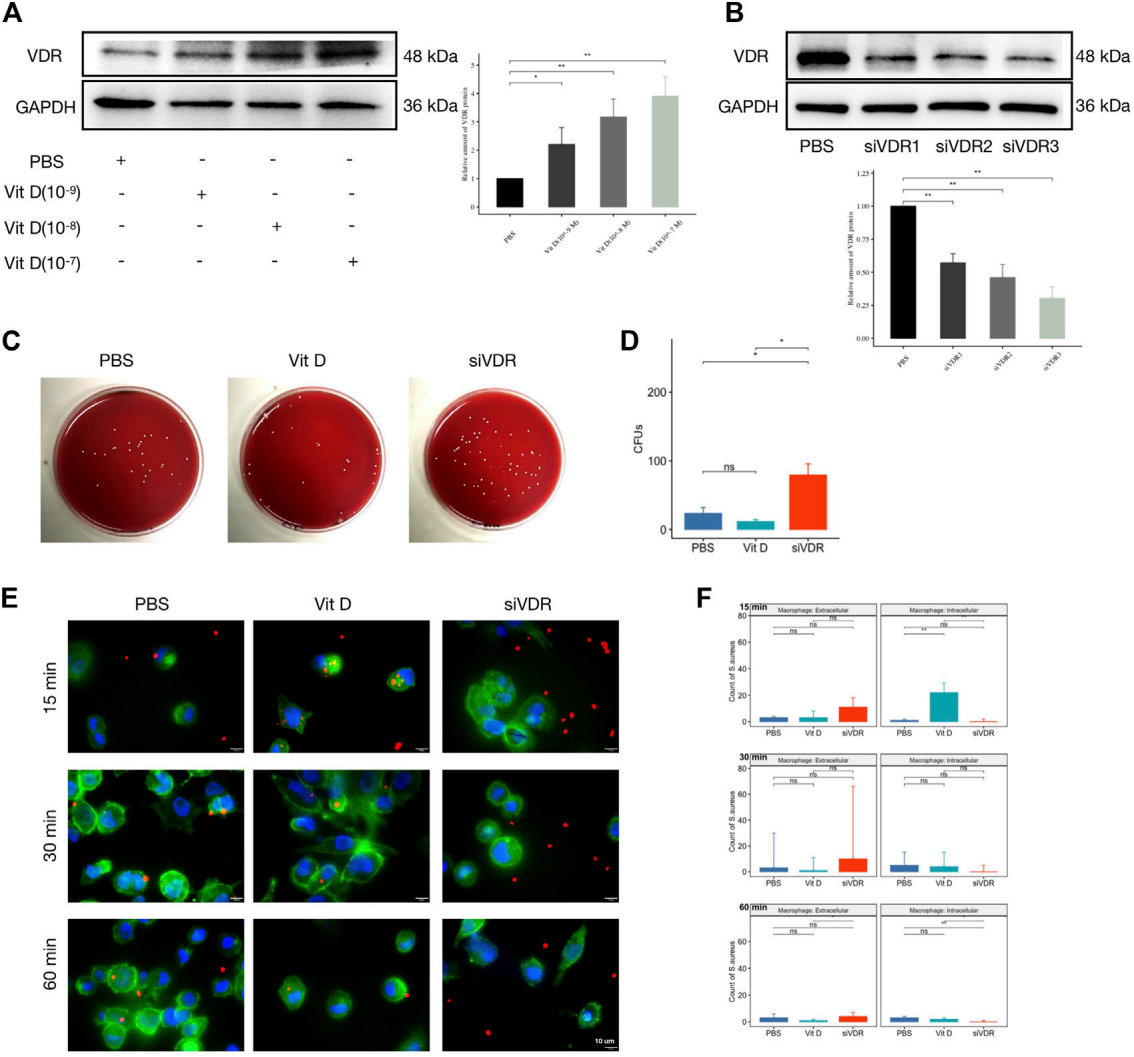
Vitamin D enhanced macrophage phagocytosis and bactericidal ability. **(A)** Active vitamin D gradient promoted VDR protein expression. **(B)** siRNAs inhibited the expression of VDR protein, and the inhibitory effect of siVDR3 was the most obvious. **(C)** Representative images after coating the plate with overexpression or knockdown of VDR protein levels. **(D)** CFUs of 1 hour after bacterial infection. **(E)** Double immunofluorescence staining for *S. aureus* (red) and F-Actin (cytoskeleton, green). Magnification, ×2,000. **(F)** Count of *S. aureus* intracellular or extracellular. “ns” for not significant, *p < 0.05, **p < 0.01, and ***p < 0.001.

In order to clarify the effect of vitamin D on macrophage functions after infection, the THP-1 cell line was first stimulated to differentiate into adherent macrophages using phorbol 12-myristate 13-acetate (PMA). The adherent macrophages were pretreated with active vitamin D or small interfering RNA (siRNA) to overexpress or knockdown the expression of the *VDR* gene and protein followed by *S. aureus* infection. As shown in Figure 3A, VDR protein expression increased with the elevation of active vitamin D concentration. Meanwhile, all of the three siRNAs inhibited the expression of VDR protein, and the inhibitory effect of siVDR3 was the most obvious (Figure 3B). Thus, we selected siVDR3 as an inhibitor of *VDR* expression in subsequent experiments.

One hour after macrophages were infected with *S. aureus*, the bacterial coating was conducted. Results showed significantly increased CFUs in the group pretreated with siVDR3, while the effect of the group pretreated with active vitamin D (Sigma-Aldrich, 10^−7^ M) was not obvious (Figures 3C,D). As shown in Figures 3E,F, active vitamin D had a greater influence on macrophage function at an early stage (15 min) after infection. In the first 15 min, macrophages pretreated with vitamin D swallowed a large number of *S. aureus*, and this effect was significantly inhibited after decreased VDR protein expression. Sixty minutes after infection, the fluorescence signal of *S. aureus* was still almost invisible in macrophages, whose VDR protein expression was inhibited (Figures 3E,F). These results suggested that the preventive effect of vitamin D on IAI may achieve by affecting the phagocytic and bactericidal functions of macrophages.

### Vitamin D Inhibited Macrophage Apoptosis Induced by *S. aureus* Associated Infection

**FIGURE 4:**
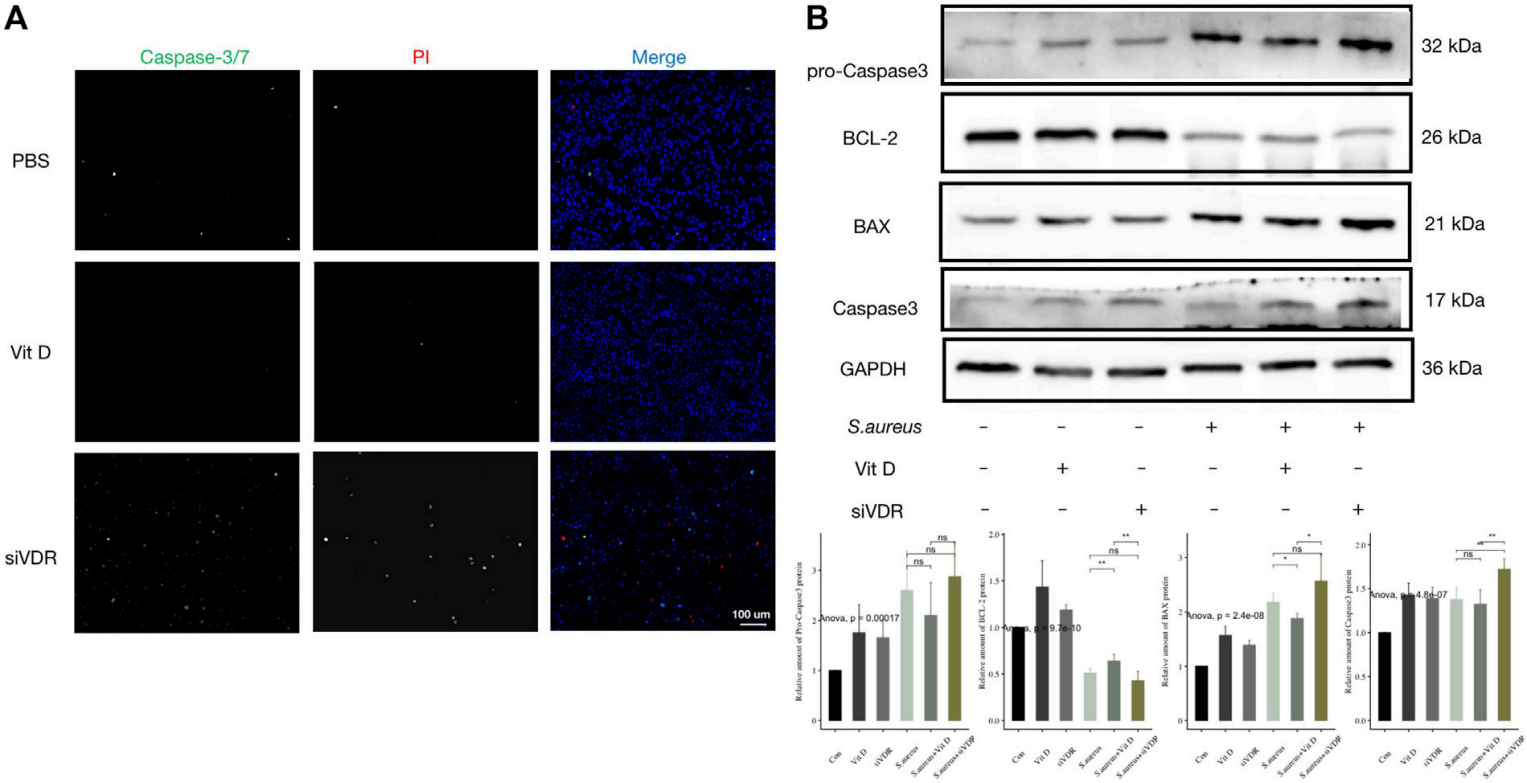
Vitamin D inhibited macrophage apoptosis induced by *S. aureus* associated infection. **(A)** Apoptosis detection, Caspase-3/7 (green) and PI (red). Magnification, ×200. **(B)** Result of western blotting for apoptosis-related proteins. “ns” for not significant, *p < 0.05, **p < 0.01, and ***p < 0.001.

Combined with the decrease of macrophages in vitamin D–deficient mice in Figure 2, and the decrease of the number of macrophages after pretreatment of siVDR in Figure 3, it is speculated that vitamin D may also affect the viability of macrophages. In order to determine whether vitamin D could affect macrophage apoptosis following infection, fluorescence staining of Caspase-3/7 and PI at 15 min after *S. aureus* infection was performed. As shown in Figure 4A, a large number of fluorescence signals of apoptosis and necrosis appeared after macrophages were pretreated with siVDR, and the application of active vitamin D could partly inhibit apoptosis and necrosis. In addition, expressions of proapoptotic-related proteins, such as BAX and caspase 3, also significantly increased in siVDR pretreated group. However, compared with PBS pretreated group, expressions of proapoptotic-related proteins were significantly decreased, while the expression of anti-apoptotic protein, BCL-2, was significantly increased in vitamin D pretreated group (Figure 4B). These results demonstrated that vitamin D effectively inhibited macrophage apoptosis following an infection caused by *S. aureus*.

### Vitamin D Inhibited Excessive Reactive Oxygen Species Expression in *S. aureus* Associated Infection

**FIGURE 5:**
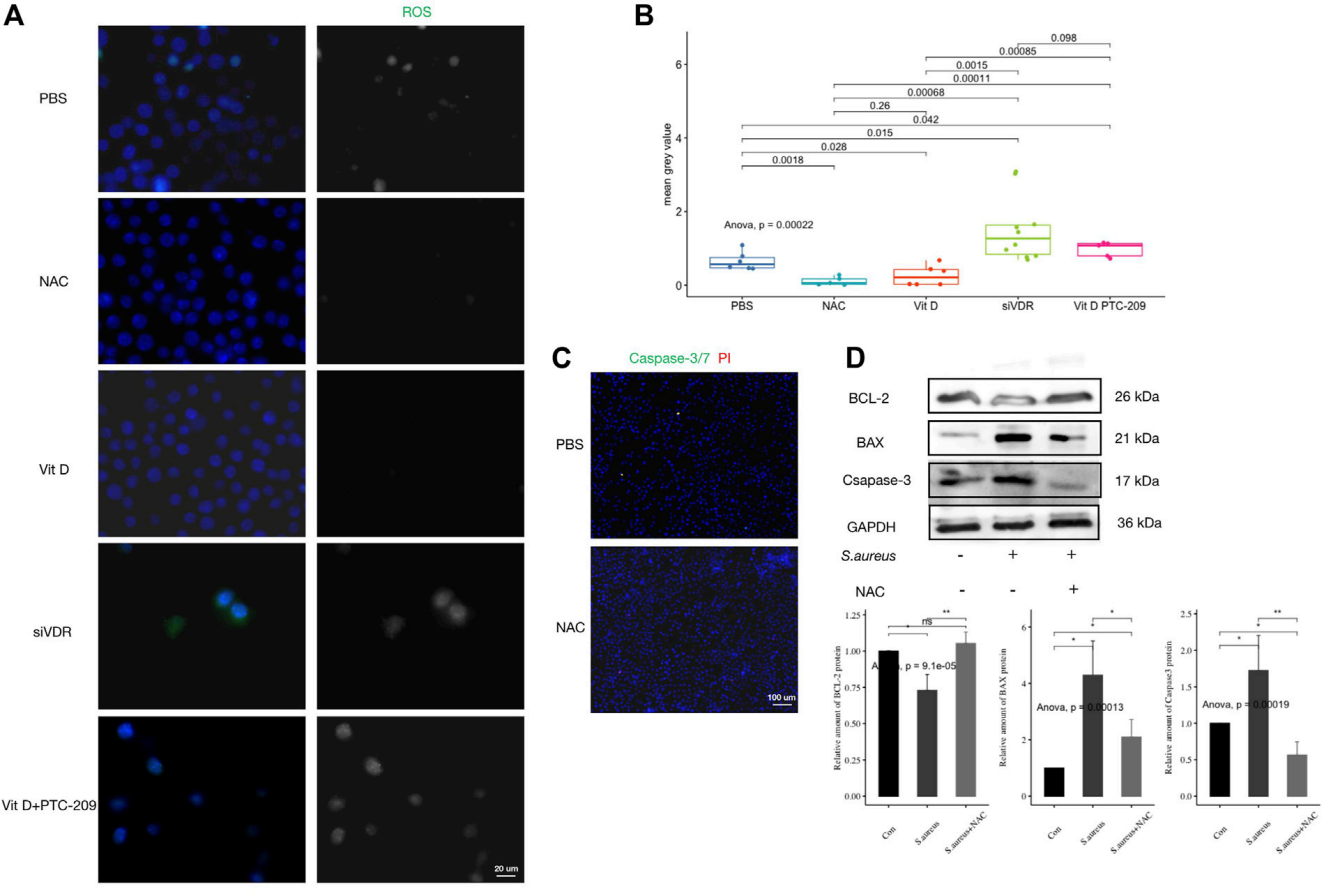
Vitamin D inhibited excessive ROS expression during *S. aureus* associated infection. **(A)** ROS generation fluorescence detection, ROS (green). Magnification, 1,000×. **(B)** Mean grey value of ROS generation. **(C)** Apoptosis detection, caspase-3/7 (green) and PI (red). Magnification, ×200. **(D)** Result of western blotting for apoptosis-related proteins. “ns” for not significant, *p < 0.05, **p < 0.01, and ***p < 0.001.

Fluorescence detection of ROS production after pretreatment with different stimuli showed that active vitamin D significantly inhibited ROS production in macrophages after infection (Figures 5A,B). The inhibitory effect of active vitamin D on ROS production was similar to that of ROS production inhibitor NAC (#A9165; Sigma-Aldrich), while pretreatment with siVDR significantly increased ROS production (Figures 5A,B). In addition, inhibition of ROS production significantly inhibited macrophages apoptosis (Figures 5C,D). These results suggested that vitamin D inhibited macrophages apoptosis by inhibiting excessive ROS production.

### Inhibition of Bmi1 Weakened the Protective Effect of Vitamin D on Macrophages

**FIGURE 6:**
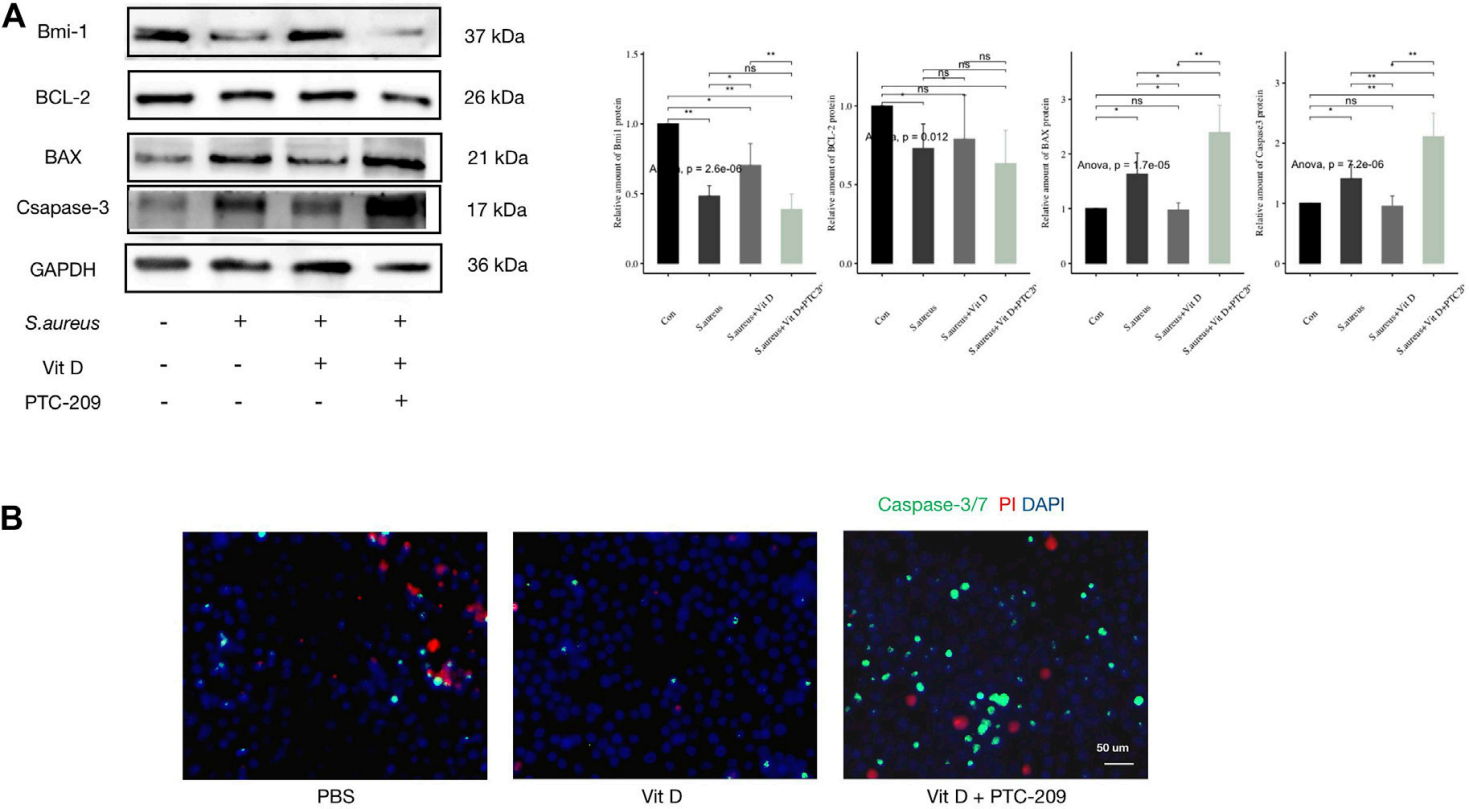
Inhibition of Bmi1 weakened the protective effect of vitamin D on macrophages. **(A)** Result of western blotting for apoptosis-related proteins. **(B)** Apoptosis detection, caspase-3/7 (green) and PI (red). Magnification, ×400. “ns” for not significant, *p < 0.05, **p < 0.01, and ***p < 0.001.

RAW264.7 cells were pretreated with active vitamin D and Bmi1 inhibitor (PTC-209) followed infection. Outcomes revealed that the inhibitory effect of active vitamin D on ROS production was significantly reversed (Figures 5A,B). Meanwhile, the use of PTC-209 significantly increased expressions of proapoptotic proteins and decreased expressions of antiapoptotic proteins (Figure 6A). In addition, PTC-209 led to the loss of such function of active vitamin D in inhibiting macrophage apoptosis. A great number of macrophages died after pretreatment with PTC-209, and the residual macrophages were mostly in the stages of apoptosis and necrosis (Figure 6B). These results indicated that vitamin D might inhibit excessive ROS production in macrophages following infection through the VDR-Bmi1 signaling pathway.

## Discussion

Many previous studies had reported positive links between *VDR* genetic variations and susceptibilities to many different inflammatory disorders, such as pulmonary tuberculosis (Xu et al., 2021), sepsis (Yang et al., 2022), and even COVID-19 (Al-Anouti et al., 2021; Apaydin et al., 2021). One of the underlying mechanisms rests with the fact that such genetic polymorphisms may affect the serum vitamin D level (Ferraz et al., 2022; Yang et al., 2022), causing different biological effects. With regard to bone infection, previous reports (Jiang et al., 2016; Zhao et al., 2022) have shown definite relationships between *VDR* gene polymorphisms and the risks of OM development, however, the detailed mechanisms remain unclear.

The present study, with a larger sample size of 398 OM patients and 368 healthy controls, suggested that *VDR* genetic polymorphisms *ApaI* and *BsmI* were associated with decreased risks of OM development, and people with AA genotype of *ApaI* and CT genotype of *BsmI* were in lower susceptibilities to OM. Additionally, patients with the above-mentioned two genotypes had relatively higher levels of vitamin D, implying that a higher vitamin D level may have a protective effect against bone infection. Then, the subsequent *in vivo* and *in vitro* experiments were designed from the perspective of different vitamin D levels on functions of the macrophages. Outcomes revealed that such a protective effect of vitamin D against OM may achieve partly by inhibiting macrophage apoptosis through inhibition of excessive ROS production *via* the VDR-Bmi1 signaling pathway.

Among the six *VDR* genetic variations, we found that rs7975232 and rs1544410 are associated with decreased risks of developing OM, with genotypes AA and CT as protective factors, respectively. Although no statistical differences were found, outcomes showed that the mutant alleles A of rs7975232 and T of rs1544410 might be protective mutations. Similarly, rs7975232 was also noted to be linked to reduced risks of developing pulmonary tuberculosis in an African population (Areeshi et al., 2017a), and hepatitis C virus infection in a Chinese population (Wu et al., 2016). As for rs1544410, it was found to be associated with progression to AIDS (Torres et al., 2010) and pulmonary tuberculosis (Areeshi et al., 2017b). With regard to another four *VDR* SNPs, although no statistical differences were found, results suggested that the genotype AG of rs2228570 might be a protective factor against OM. Therefore, further studies with a larger sample size are warranted to obtain more accurate conclusions. The current study failed to find any positive relationships between *TaqI*, *GATA*, or *Cdx-2* and the risks of developing OM in this Chinese cohort.

With respect to the relationships between *VDR* SNPs and serum vitamin D levels, we found that the mean level of vitamin D among OM patients with protective genotypes (AA of *ApaI* and CT of *BsmI*) was relatively higher than other corresponding genotypes. This suggests that the potential mechanisms of *VDR* SNPs involved in the pathogenesis of OM may achieve partly *via* their influences on vitamin D levels, which was similar to several previous investigations. Ferraz et al. (Ferraz et al., 2022) observed that *VDR* variants may participate in the development of type 1 diabetes mellitus in a Brazilian population by influencing 25(OH)D level. Similarly, Awasthi et al. (2021) indicated that *VDR* genetic variation, associated with the risk of developing community-acquired pneumonia, also affected peripheral vitamin D levels. It is interesting that, aside from *ApaI* and *BsmI*, patients with AG genotype of rs2228570 had a significantly higher mean level of vitamin D than those with AA and GG genotypes, which was inconsistent with the previous finding that AG might be related to a reduced risk of OM development.

As mentioned previously, *VDR* genetic variations may involve in the development of OM by affecting peripheral vitamin D levels. Meanwhile, growing evidence has revealed a positive link between vitamin D and the occurrence of bone and joint infections. In 2014, Maier et al. (2014) found a high frequency of vitamin D deficiency in patients being received primary arthroplasty and those with aseptic joint prosthetic loosening and PJI, with vitamin D deficiency being more severe in PJI patients. Later in 2017, Hegde et al. (2017b) conducted a mouse model of PJI and observed that vitamin D deficiency resulted in increased bacterial burden and neutrophil infiltration, which can be reversed with preoperative repletion of vitamin D. In 2018 and 2019, preoperative low vitamin D level has been recognized and listed as a risk factor of PJI (Alamanda and Springer, 2018; Alamanda and Springer, 2019). However, detailed mechanisms of vitamin D in the pathogenesis of osteoarticular infections remain largely unclear. Our subsequent experiments were designed from the perspective of the influences of different vitamin D levels on functions of the immune cells.

It is known that macrophages act as one of the primary immune cells after pathogenic microorganisms invade the human body, we focused on the effects of vitamin D on macrophage functions, with different interventional factors pretreating macrophages followed by infection with *S. aureus*. Outcomes showed that active vitamin D promoted phagocytosis of macrophages to *S. aureus* at an early stage following up-regulation of the VDR protein expression. While with the decrease of VDR expression, its bactericidal ability reduced. In addition, as the vitality of immune cells is one of the key factors accounting for bactericidal ability, we noted down-regulation of VDR expression significantly inhibited macrophages viability after the bacterial invasion and its apoptosis obviously increased. This may be one of the reasons for the decreased number of local macrophages in the vitamin D–deficient IAI mice.

Previous studies have indicated that macrophages infected with *S. aureus* induced increased intracellular ROS production and endoplasmic reticulum (ER) stress response (Kumar et al., 2020). ROS is an important material basis for macrophages to kill invading bacteria (Di Cara et al., 2017). However, excessive ROS production is also an initiating factor of apoptosis (Roca et al., 2019). Previous investigations pointed out that active vitamin D protected renovascular function in hypertension by reducing oxidative stress (Dong et al., 2012). Therefore, we inferred that the inhibitory effect of vitamin D on macrophage apoptosis might achieve by reducing excessive ROS production during bacterial infection. The current results also confirmed our conjecture that excessive ROS production accelerated the self-destruction process of macrophages, and activation of active vitamin D-VDR signal inhibited ROS production, and then improved the phagocytic and bactericidal functions of macrophages by reducing apoptosis.

As for the mechanisms by which VDR reduces ROS generation, a recent study reported that active vitamin D inhibited ROS production in mesenchymal stem cells (MSCs) by activating the VDR-Bmi1 signal pathway, and then inhibited cell oxidative stress, DNA damage, as well as cellular senescence, and finally achieved the effect of anti-osteoporosis (Sun et al., 2020). Thus, we speculated that the VDR-Bmi1 signaling pathway of active vitamin D may also play a role during bacterial infection. In this experiment, we found that when active vitamin D was combined with Bmi1 inhibitor, the improvement effect of active vitamin D on macrophage functions disappeared. After *S. aureus* infected cells, apoptosis and level of ROS production of macrophages pretreated with Bmi1 inhibitor were significantly up-regulated. Therefore, we considered that during infection, active vitamin D might inhibit excessive ROS through the VDR-Bmi1 signaling pathway.

The present study also has limitations. First, as OM is a disorder of high heterogeneity, the occurrence of which is linked to complex interactions between extrinsic and intrinsic factors, our study only analyzed its pathogenesis from the perspective of genetic predisposition, which is more or less with bias. Second, we only analyzed the potential influences of vitamin D on macrophage functions and states during infection. It is known that bacterial infection is a complex immune response process, which contains combined actions of multiple cells, such as neutrophils, natural killer cells, and plasma cells. Whether vitamin D affects the functions of other immune cells needs to be further explored. Third, although both *in vivo* and *in vitro* experiments were conducted to investigate the potential role of vitamin D in the development of OM, there still existed flaws. For example, the signal pathway VDR-Bmi1-ROS was conducted and certified *via in-vitro* experiments only, which requires to be evaluated in animal studies. In addition, we did not detect vitamin D levels in animals used for the experiment as in a previous study (Hegde et al., 2017b), which confirmed that diet and intraperitoneal vitamin D injection can significantly increase the serum vitamin D level in mice, which is another limitation.

## Conclusion

In summary, *ApaI* and *BsmI* are associated with a decreased risk of OM development, with AA and CT genotypes as protective factors, respectively. Patients with the mentioned two genotypes had relatively higher levels of vitamin D as compared with other corresponding genotypes, implying that the protective role of VDR genetic variations may be achieved partly by influencing peripheric vitamin D levels. Such a protective role may be achieved partly by inhibiting macrophage apoptosis through the inhibition of excessive ROS production *via* the VDR-Bmi1 signaling pathway.

## Data Availability

The original contributions presented in the study are included in the article/Supplementary Material; further inquiries can be directed to the corresponding authors.

## References

[B1] Al-Anouti F. Mousa M. Karras S. N. Grant W. B. Alhalwachi Z. Abdel-Wareth L. (2021). Associations between Genetic Variants in the Vitamin D Metabolism Pathway and Severity of COVID-19 Among UAE Residents. Nutrients 13 (11), 3680. PubMed PMID: WOS:000728695600001. 10.3390/Nu13113680 34835935 PMC8625365

[B2] Alamanda V. K. Springer B. D. (2018). Perioperative and Modifiable Risk Factors for Periprosthetic Joint Infections (PJI) and Recommended Guidelines. Curr. Rev. Musculoskelet. Med. 11 (3), 325–331. PubMed PMID: 29869135; PubMed Central PMCID: PMC6105493. 10.1007/s12178-018-9494-z 29869135 PMC6105493

[B3] Alamanda V. K. Springer B. D. (2019). The Prevention of Infection. bone & Jt. J. 101-B, 3–9. (1_Supple_A)Epub 2019/01/17PubMed PMID: 30648488. 10.1302/0301-620x.101b1.bjj-2018-0233.r1 30648488

[B4] Alves De Souza C. Queiroz Alves De Souza A. Queiroz Alves De Souza M. d. S. Dias Leite J. A. Silva De Morais M. Barem Rabenhorst S. H. (2017). A Link between Osteomyelitis and IL1RN and IL1B Polymorphisms-A Study in Patients from Northeast Brazil. Acta Orthop. 88 (5), 556–561. Epub 2017/07/07PubMed PMID: 28682145; PubMed Central PMCID: PMCPmc5560221. 10.1080/17453674.2017.1348439 28682145 PMC5560221

[B5] Apaydin T. Polat H. Dincer Yazan C. Ilgin C. Elbasan O. Dashdamirova S. (2021). Effects of Vitamin D Receptor Gene Polymorphisms on the Prognosis of COVID‐19. Clin. Endocrinol. 96, 819–830. PubMed PMID: 34919268. 10.1111/cen.14664 34919268

[B6] Areeshi M. Y. Mandal R. K. Wahid M. Dar S. A. Jawed A. Lohani M. (2017). Vitamin D Receptor ApaI (Rs7975232) Polymorphism Confers Decreased Risk of Pulmonary Tuberculosis in Overall and African Population, but Not in Asians: Evidence from a Meta-Analysis. Ann. Clin. Lab. Sci. 47 (5), 628–637. Epub 2017/10/27. PubMed PMID: 29066494. 29066494

[B7] Areeshi M. Y. Mandal R. K. Dar S. A. Alshahrani A. M. Ahmad A. Jawed A. (2017). A Reappraised Meta-Analysis of the Genetic Association between Vitamin D Receptor BsmI (Rs1544410) Polymorphism and Pulmonary Tuberculosis Risk. Biosci. Rep. 37, 37. PubMed PMID: WOS:000404246900037. 10.1042/Bsr20170247 PMC5463263 28533426

[B8] Arshad Z. Lau E. J.-S. Aslam A. Thahir A. Krkovic M. (2021). Management of Chronic Osteomyelitis of the Femur and Tibia: a Scoping Review. EFORT Open Rev. 6 (9), 704–715. PubMed PMID: 34667641; PubMed Central PMCID: PMC8489473. 10.1302/2058-5241.6.200136 34667641 PMC8489473

[B9] Awasthi N. Awasthi S. Pandey S. (2021). Role of VDR Gene Polymorphisms with Community Acquired Pneumonia in North Indian Children: a Case-Control Study. Int. J. Mol. Epidemiol. Genet. 12 (1), 1–8. PubMed PMID: 33859782; PubMed Central PMCID: PMC8044708. 33859782 PMC8044708

[B10] Bernthal N. M. Stavrakis A. I. Billi F. Cho J. S. Kremen T. J. Simon S. I. (2010). A Mouse Model of Post-arthroplasty *Staphylococcus aureus* Joint Infection to Evaluate *In Vivo* the Efficacy of Antimicrobial Implant Coatings. PLoS One 5 (9), e12580. PubMed PMID: 20830204; PubMed Central PMCID: PMC2935351. 10.1371/journal.pone.0012580 20830204 PMC2935351

[B11] Birt M. C. Anderson D. W. Bruce Toby E. Wang J. (2017). Osteomyelitis: Recent Advances in Pathophysiology and Therapeutic Strategies. J. Orthop. 14 (1), 45–52. PubMed PMID: 27822001; PubMed Central PMCID: PMC5090239. 10.1016/j.jor.2016.10.004 27822001 PMC5090239

[B12] Di Cara F. Sheshachalam A. Braverman N. E. Rachubinski R. A. Simmonds A. J. (2017). Peroxisome-Mediated Metabolism Is Required for Immune Response to Microbial Infection. Immunity 47 (1), 93–106. e7PubMed PMID: 28723556. 10.1016/j.immuni.2017.06.016 28723556

[B13] Dong J. Wong S. L. Lau C. W. Lee H. K. Ng C. F. Zhang L. (2012). Calcitriol Protects Renovascular Function in Hypertension by Down-Regulating Angiotensin II Type 1 Receptors and Reducing Oxidative Stress. Eur. heart J. 33 (23), 2980–2990. PubMed PMID: 22267242. 10.1093/eurheartj/ehr459 22267242

[B14] Ferraz R. S. Silva C. S. Cavalcante G. C. de Queiroz N. N. M. Felício K. M. Felício J. S. (2022). Variants in the VDR Gene May Influence 25(OH)D Levels in Type 1 Diabetes Mellitus in a Brazilian Population. Nutrients 14 (5), 1010. PubMed PMID: WOS:000768922300001. 10.3390/Nu14051010 35267984 PMC8912721

[B15] Hegde V. Dworsky E. M. Stavrakis A. I. Loftin A. H. Zoller S. D. Park H. Y. (2017). Single-Dose, Preoperative Vitamin-D Supplementation Decreases Infection in a Mouse Model of Periprosthetic Joint Infection. J. Bone Jt. Surg. 99 (20), 1737–1744. PubMed PMID: WOS:000418579100011. 10.2106/Jbjs.16.01598 PMC6948832 29040128

[B16] Hegde V. Dworsky E. M. Stavrakis A. I. Loftin A. H. Zoller S. D. Park H. Y. (2017). Single-Dose, Preoperative Vitamin-D Supplementation Decreases Infection in a Mouse Model of Periprosthetic Joint Infection. J. Bone Jt. Surg. 99 (20), 1737–1744. PubMed PMID: 29040128. 10.2106/JBJS.16.01598 PMC6948832 29040128

[B17] Hung C.-H. Ko J.-Y. Liao P.-S. Yeh C.-W. Hsu C.-C. Lin M.-C. (2020). Epidemiology of Fatal/non-Fatal Suicide Among Patients with Chronic Osteomyelitis (COM): a Nationwide Population-Based Study. J. Int. Med. Res. 48 (6), 030006052091923. PubMed PMID: WOS:000548783500001. 10.1177/0300060520919238 PMC7331771 32605410

[B18] Jiang N. Li S.-y. Ma Y.-f. Hu Y.-j. Lin Q.-r. Yu B. (2020). Associations between Interleukin Gene Polymorphisms and Risks of Developing Extremity Posttraumatic Osteomyelitis in Chinese Han Population. Mediat. Inflamm. 2020, 1–9. PubMed PMID: WOS:000536143300002. 10.1155/2020/3278081 PMC7222541 32454789

[B19] Jiang N. Li S.-y. Zhang P. Yu B. (2020). Clinical Characteristics, Treatment, and Prognosis of Squamous Cell Carcinoma Arising from Extremity Chronic Osteomyelitis: a Synthesis Analysis of One Hundred and Seventy Six Reported Cases. Int. Orthop. (SICOT) 44 (11), 2457–2471. PubMed PMID: 32705317. 10.1007/s00264-020-04737-0 32705317

[B20] Jiang N. Zhao X.-q. Qin C.-h. Hu Y.-j. Wang L. Xie G.-p. (2016). Association of Vitamin D Receptor Gene TaqI, BsmI, FokI and ApaI Polymorphisms and Susceptibility to Extremity Chronic Osteomyelitis in Chinese Population. Injury 47 (8), 1655–1660. Epub 2016/06/23PubMed PMID: 27329975. 10.1016/j.injury.2016.06.005 27329975

[B21] Kong Q. Jin Y. Yan S. Wang Y. Zhao J. Feng Z. (2017). Examining the Association of MMP-1 Gene −1607 (2G/1G) and −519 (A/G) Polymorphisms with the Risk of Osteomyelitis. Medicine 96 (42), e4969. PubMed PMID: 29049163; PubMed Central PMCID: PMC5662329. 10.1097/MD.0000000000004969 29049163 PMC5662329

[B22] Kumar A. Singh P. K. Zhang K. Kumar A. (2020). Toll‐like Receptor 2 (TLR2) Engages Endoplasmic Reticulum Stress Sensor IRE1α to Regulate Retinal Innate Responses in *Staphylococcus aureus* Endophthalmitis. FASEB J. 34 (10), 13826–13838. PubMed PMID: 32813318. 10.1096/fj.202001393R 32813318 PMC8033405

[B23] Maier G. S. Horas K. Seeger J. B. Roth K. E. Kurth A. A. Maus U. (2014). Is There an Association between Periprosthetic Joint Infection and Low Vitamin D Levels? Int. Orthop. (SICOT) 38 (7), 1499–1504. PubMed PMID: 24737149; PubMed Central PMCID: PMC4071483. 10.1007/s00264-014-2338-6 PMC4071483 24737149

[B24] McNally M. Govaert G. Dudareva M. Morgenstern M. Metsemakers W.-J. (2020). Definition and Diagnosis of Fracture-Related Infection. EFORT Open Rev. 5 (10), 614–619. Epub 2020/11/19PubMed PMID: 33204503; PubMed Central PMCID: PMCPMC7608516. 10.1302/2058-5241.5.190072 33204503 PMC7608516

[B25] Pérez-Is L. Ocaña M. G. Montes A. H. Carton J. A. Álvarez V. Meana Á. (2019). The N125S Polymorphism in the Cathepsin G Gene (Rs45567233) Is Associated with Susceptibility to Osteomyelitis in a Spanish Population. Plos One 14 (10), e0220022. PubMed PMID: WOS:000532631800008. 10.1371/journal.pone.0220022 31647805 PMC6812796

[B26] Roca F. J. Whitworth L. J. Redmond S. Jones A. A. Ramakrishnan L. (2019). TNF Induces Pathogenic Programmed Macrophage Necrosis in Tuberculosis through a Mitochondrial-Lysosomal-Endoplasmic Reticulum Circuit. Cell. 178 (6), 1344–1361. e11PubMed PMID: 31474371. 10.1016/j.cell.2019.08.004 31474371 PMC6736209

[B27] Singh A. K. Prakash S. Garg R. K. Jain P. Kumar R. Jain A. (2021). Polymorphisms in Vitamin D Receptor, Toll-like Receptor 2 and Toll-like Receptor 4 Genes Links with Dengue Susceptibility. Bioinformation 17 (4), 506–513. PubMed PMID: 34602778; PubMed Central PMCID: PMC8450152. 10.6026/97320630017506 34602778 PMC8450152

[B28] Smeltzer M. S. Thomas J. R. Hickraon S. G. Skinner R. A. Nelson C. L. Griffith D. (1997). Characterization of a Rabbit Model of Staphylococcal Osteomyelitis. J. Orthop. Res. 15 (3), 414–421. Epub 1997/05/01PubMed PMID: 9246088. 10.1002/jor.1100150314 9246088

[B29] Sun H. Qiao W. Cui M. Yang C. Wang R. Goltzman D. (2020). The Polycomb Protein Bmi1 Plays a Crucial Role in the Prevention of 1,25(OH) 2 D Deficiency‐Induced Bone Loss. J. Bone Min. Res. 35 (3), 583–595. PubMed PMID: 31725940. 10.1002/jbmr.3921 31725940

[B30] Torres C. Sanchez-de-la-Torre M. Garcia-Moruja C. Carrero A. Trujillo M. Fibla J. (2010). Immunophenotype of Vitamin D Receptor Polymorphism Associated to Risk of HIV-1 Infection and Rate of Disease Progression. Chr 8 (6), 487–492. PubMed PMID: 20642435. 10.2174/157016210793499330 20642435

[B31] Tseng C.-H. Huang W.-S. Muo C.-H. Chang Y.-J. Kao C.-H. (2014). Increased Depression Risk Among Patients with Chronic Osteomyelitis. J. Psychosomatic Res. 77 (6), 535–540. PubMed PMID: WOS:000347741700015. 10.1016/j.jpsychores.2014.09.008 25258357

[B32] Wang Y. Cheng L. I. Helfer D. R. Ashbaugh A. G. Miller R. J. Tzomides A. J. (2017). Mouse Model of Hematogenous Implant-Related *Staphylococcus aureus* Biofilm Infection Reveals Therapeutic Targets. Proc. Natl. Acad. Sci. U.S.A. 114 (26), E5094–E102. PubMed PMID: 28607050; PubMed Central PMCID: PMC5495257. 10.1073/pnas.1703427114 28607050 PMC5495257

[B33] Wu M. Yue M. Huang P. Zhang Y. Xie C. Yu R. (2016). Vitamin D Level and Vitamin D Receptor Genetic Variations Contribute to HCV Infection Susceptibility and Chronicity in a Chinese Population. Infect. Genet. Evol. 41, 146–152. PubMed PMID: 27063396. 10.1016/j.meegid.2016.03.032 27063396

[B34] Xu S. Xie X. Jiao L. Bai H. Wu X. Ying J. (2021). Association Analysis of Pulmonary Tuberculosis and Vitamin D Receptor Gene Polymorphisms of Han Population in Western China. Microb. Pathog. 161 (Pt A), 105190. PubMed PMID: 34619312. 10.1016/j.micpath.2021.105190 34619312

[B35] Yang X. Ru J. Li Z. Jiang X. Fan C. (2022). Lower Vitamin D Levels and VDR FokI Variants Are Associated with Susceptibility to Sepsis: a Hospital-Based Case-Control Study. Biomarkers 27 (2), 188–195. and susceptibility to chemicalsPubMed PMID: 35001797. 10.1080/1354750X.2021.2024598 35001797

[B36] Zhao X.-q. Chen K. Wan H.-y. He S.-y. Qin H.-j. Yu B. (2022). Vitamin D Receptor Genetic Variations May Associate with the Risk of Developing Late Fracture-Related Infection in the Chinese Han Population. J. Immunol. Res. 2022, 1–8. PubMed PMID: WOS:000770861200001. 10.1155/2022/9025354 PMC8886694 35242885

